# Personalized Nutrition Through the Gut Microbiome in Metabolic Syndrome and Related Comorbidities

**DOI:** 10.3390/nu18020290

**Published:** 2026-01-16

**Authors:** Julio Plaza-Diaz, Lourdes Herrera-Quintana, Jorge Olivares-Arancibia, Héctor Vázquez-Lorente

**Affiliations:** 1 Departament de Bioquímica i Biotecnologia, ANUT-DSM (Alimentaciò, Nutrició Desenvolupament i Salut Mental), Universitat Rovira i Virgili, 43201 Reus, Spain; 2 Institut d’Investigació Sanitària Pere Virgili (IISPV), 43204 Reus, Spain; 3 Biomedical Research Networking Center for Physiopathology of Obesity and Nutrition (CIBERObn), Institute of Health Carlos III, 28029 Madrid, Spain; 4 School of Health Sciences, Universidad Internacional de La Rioja, 26006 Logroño, Spain; 5 Laboratory of Cellular and Molecular Gerontology, Precision Nutrition and Aging, Madrid Institute for Advanced Studies—IMDEA Nutrition, CEI UAM+CSIC, 28049 Madrid, Spain; lourdes.herrera@nutricion.imdea.org; 6 AFySE Group, Research in Physical Activity and School Health, School of Physical Education, Faculty of Education, Universidad de Las Américas, Santiago 7500975, Chile; jorge.olivares.ar@gmail.com

**Keywords:** personalized nutrition, gut microbiome, metabolic syndrome, insulin resistance, dysbiosis, short-chain fatty acids, prebiotics, probiotics, precision nutrition

## Abstract

**Background**: Metabolic syndrome, a clinical condition defined by central obesity, impaired glucose regulation, elevated blood pressure, hypertriglyceridemia, and low high-density lipoprotein cholesterol across the lifespan, is now a major public health issue typically managed with lifestyle, behavioral, and dietary recommendations. However, “one-size-fits-all” recommendations often yield modest, heterogeneous responses and poor long-term adherence, creating a clinical need for more targeted and implementable preventive and therapeutic strategies. **Objective**: To synthesize evidence on how the gut microbiome can inform precision nutrition and exercise approaches for metabolic syndrome prevention and management, and to evaluate readiness for clinical translation. **Key findings**: The gut microbiome may influence cardiometabolic risk through microbe-derived metabolites and pathways involving short-chain fatty acids, bile acid signaling, gut barrier integrity, and low-grade systemic inflammation. Diet quality (e.g., Mediterranean-style patterns, higher fermentable fiber, or lower ultra-processed food intake) consistently relates to more favorable microbial functions, and intervention studies show that high-fiber/prebiotic strategies can improve glycemic control alongside microbiome shifts. Physical exercise can also modulate microbial diversity and metabolic outputs, although effects are typically subtle and may depend on baseline adiposity and sustained adherence. Emerging “microbiome-informed” personalization, especially algorithms predicting postprandial glycemic responses, has improved short-term glycemic outcomes compared with standard advice in controlled trials. Targeted microbiome-directed approaches (e.g., *Akkermansia muciniphila*-based supplementation and fecal microbiota transplantation) provide proof-of-concept signals, but durability and scalability remain key limitations. **Conclusions:** Microbiome-informed personalization is a promising next step beyond generic guidelines, with potential to improve adherence and durable metabolic outcomes. Clinical implementation will require standardized measurement, rigorous external validation on clinically meaningful endpoints, interpretable decision support, and equity-focused evaluation across diverse populations.

## 1. Introduction

Metabolic syndrome (MetS) is a clinical condition defined by the combination of central obesity, hypertension, impaired glucose, elevated triglycerides, and low high-density lipoprotein cholesterol [1]. MetS substantially amplifies the risk of multiple complications for a large proportion of global morbidity, disability, and health-care costs [2,3,4,5]. Current estimates suggest that up to one in three adults may meet criteria for MetS, underscoring its importance as a major non-communicable disease priority [6].

Changes in diet and physical exercise remain the cornerstone of MetS prevention and management in clinical practice and public health guidelines [7]. Diet recommendations are focused on energy restriction, weight loss, and improvement in diet quality, with particular attention to cardiometabolic dietary patterns, including the Mediterranean and DASH diets [8,9,10]. Of note, clinical trials and cohort studies based on diet and physical exercise have shown important benefits using a general approach [11,12,13]. However, “one-size-fits-all” guidelines have frequently yielded modest and heterogeneous responses at the individual level, and long-term adherence is often poor [11]. In routine care, this combination of variable response and poor adherence translates into repeated cycles of weight regain and persistent cardiometabolic risk. This highlights a practical clinical gap: clinicians need implementable strategies to match dietary and lifestyle prescriptions to the patients most likely to benefit and adhere to them [14]. Recent studies have shown inter-individual variability in postprandial glycemic and lipemic responses to standardized meals, even among individuals with similar clinical characteristics, highlighting substantial biological heterogeneity and suggesting that generalized dietary prescriptions may be suboptimal for many patients with or at risk of MetS [15,16,17]. Therefore, personalization is clinically relevant not only as a mechanistic refinement, but also to improve the “fit” of recommendations to patient biology and preferences, potentially strengthening adherence and yielding more durable improvements in glycemic control, lipemia, and other MetS components [18].

The gut microbiome has emerged as a key biological candidate to explain some of this variability and to refine weight-loss lifestyle interventions for cardiometabolic health [19]. The intestinal tract harbors a complex, dynamic community of bacteria, Archaea, viruses, and fungi whose collective genomes and metabolic activities profoundly influence host physiology [20,21]. Beyond their classical roles in nutrient metabolism and energy harvest, gut microbes contribute to the biotransformation of dietary components, short-chain fatty acids (SCFAs), and other relevant bioactive metabolites production [22]. In addition, they influence the regulation of bile acid (BA) pools and signaling, modulation of intestinal barrier integrity and immune tone, and crosstalk with endocrine and neural pathways [23,24]. Perturbations in microbiome diversity and structure (“dysbiosis”)—often characterized by reduced diversity and depletion of SCFA-producing taxa—have been repeatedly associated with obesity and MetS-related phenotypes. Although causal relationships and the directionality of these associations remain areas of active investigation to date [25].

Personalized nutrition broadly refers to the tailoring of dietary recommendations to individual characteristics, including clinical and metabolic profiles, genetic background, microbiome features, and behavioral patterns and preferences, with the goal of optimizing human health outcomes [26]. Advances in high-throughput “omics” technologies, including host genomics, metabolomics, and microbiome-focused metagenomics and metatranscriptomics, along with deep metabolic phenotyping (e.g., continuous glucose monitoring, postprandial testing) and digital health tools (wearables, mobile applications, machine-learning algorithms), now enable the collection and integration of large-scale, person-specific data streams [27].

Recent studies have shown that models incorporating data from gut microbiome, together with clinical, lifestyle, and dietary approaches, can improve the prediction of individual postprandial glycemic responses to meals [28,29]. In some cases, these models have been used to guide personalized dietary interventions that improve short-term glycemic control compared with standard approaches [30]. In parallel, more recent trials have extended these approaches to broader cardiometabolic endpoints, using multi-omics and digital phenotyping to design app-based personalized dietary programs for individuals at increased metabolic risk [31,32]. Nevertheless, most available studies were short term, involved relatively small and selected cohorts, and their generalizability and long-term clinical impact remain uncertain.

This review aims to inform the development of precision strategies for the prevention and management of MetS. Alongside diet and physical activity, modulation of the gut microbiome is increasingly recognized as a central component of MetS care, with beneficial effects on insulin sensitivity and chronic low-grade systemic inflammation. Here, we describe the diet–gut microbiome–host axis in MetS and the key mechanistic pathways through which microbial activity influences host metabolism. We discuss personalized nutrition and exercise as foundational elements of lifestyle management and summarize intervention studies. Finally, we evaluate the potential and the current limitations of integrating microbiome profiles with clinical, metabolic, and fitness measures to support individualized lifestyle recommendations.

## 2. Concept and Tools of Personalized Nutrition

### 2.1. Definitions and Frameworks

Population-based dietary guidelines are designed to improve human health at scale by targeting the “average” person within broad life-stage or sex categories [33]. This public-health logic is fundamentally different from personalized approaches, which start from the premise that interindividual variability in physiology, behaviors, and contexts, meaningfully shapes dietary response [34]. In this space, a widely used definition frames personalized nutrition “as the use of information on individual characteristics to deliver targeted advice, products, or services that facilitate sustained, health-relevant dietary change” [35].

Between generic guidelines and fully individualized prescriptions lies stratified nutrition, which tailors recommendations to subgroups (e.g., defined by phenotype, baseline risk, or other measurable characteristics) rather than “*n* = 1” designs, an increasingly relevant concept as dietary guidance evolves toward identifying population segments with distinct needs and response profiles. In parallel, precision nutrition is commonly used to emphasize the integration of multi-layer biological and behavioral data (multi-omics, clinical phenotypes, microbiome features, and digital measures) with analytic methods to generate recommendations that are more granular, dynamic, and potentially adaptative over time [35,36].

Operationally, “levels of personalization” can be organized as phenotype-based (clinical and biochemical traits), genotype-based (nutrigenetic/nutrigenomic information where evidence supports differential response), microbiome-based (composition and functional capacity), and digital/behavioral tailoring (preferences, barriers, context, and real-time behavioral/physiological signals) [37,38]. Importantly, the field’s credibility depends on demonstrating that these added layers improve prediction and, crucially, translate into durable behavior change and better outcomes; large pragmatic trials such as Food4Me [39] provide evidence that personalized advice can improve dietary behaviors compared with conventional guidance, while also underscoring the need for rigorous evaluation across populations and endpoints [39].

### 2.2. Data Layers in Precision Nutrition

A precision nutrition architecture typically begins with host factors, spanning genetics/epigenetics and other omics, alongside clinical phenotypes that anchor recommendations in cardiometabolic risk, adiposity, and related traits [40]. Contemporary frameworks emphasize that these biomedical layers should not be treated in isolation [18]. They are most informative when combined with behavioral signatures and contextual determinants that influence both exposure (diet) and response (adherence and physiology) [18].

Microbiome adds an additional set of data layers that move from “who is there” to “what they can do” and “what they are doing.” In practice, 16S rRNA profiling provides taxonomic structure but limited functional resolution [41]. However, shotgun metagenomics improves taxonomic breadth and enables pathway-level functional inference; comparative work shows that 16S rRNA [42]. Metatranscriptomics extends this further by quantifying microbial gene expression (activity), and metabolomics captures downstream host-microbe co-metabolites that are often closest to mechanism and phenotype [43].

Finally, digital tools and artificial intelligence (AI) enable high-frequency, real-world measurement and iterative feedback [44]. They provide the substrate for machine-learning models that predict postprandial responses [45]. Evidence demonstrated that integrating clinical features, lifestyle data, and microbiome information can improve prediction of individualized glycemic (and broader metabolic) responses [45]. At the same time, systematic reviews highlight that model performance, generalizability, and clinical utility depend on transparent validation and careful feature selection, particularly when translating from controlled cohorts to diverse populations and settings.

## 3. Diet–Gut Microbiome–Host Axis in Metabolic Syndrome

### 3.1. Core Microbiome Alterations in Metabolic Syndrome and Obesity

Several observational and metagenomic studies indicates that obesity and MetS are accompanied by characteristic, albeit heterogeneous, alterations in gut microbiome composition [46,47,48]. A frequently reported feature is reduced microbial α-diversity, often interpreted as a loss of ecological resilience and functional redundancy, depending of the α-diversity metrics used [49].

With the intention to create a unique variable for disease, including obesity, the early work proposed an increased *Bacillota*/*Bacteroidota* ratio as a hallmark of obesity [50]. However, subsequent studies across different populations and sequencing platforms have yielded inconsistent results, with some reporting no differences or even opposite trends [50,51]. These discrepancies underscore that simple phylum-level metrics are unlikely to capture the complexity of obesity- and MetS-associated dysbiosis and that taxonomic shifts are context-dependent, influenced by diet, medication use, geography, and host genetics [52].

Regarding to species taxa, several recurrent patterns have been described [53,54,55,56], although not universally replicated. Results from several cohorts have shown that individuals living with obesity or MetS often show depletion of SCFA-producing species, such as *Faecalibacterium prausnitzii* [57] and certain *Roseburia* and *Eubacterium* species. In contrast, they also shown enrichment of genera like *Collinsella*, *Blautia*, or *Prevotella* [53,54,55,56]. These gut microbiome compositional shifts are related to adverse metabolic traits, including key components of MetS [58,59]. For example, microbiome profiles are enriched in lipopolysaccharide (LPS) biosynthesis pathways or branched-chain amino acid (BCAA) production. BA-modifying enzymes have also been associated with higher HOMA-IR, increased triglycerides, hepatic steatosis, and markers of vascular risk [58,59].

However, it is increasingly recognized that there is no single “obese” or “MetS” microbiome [60]. MetS microbial signature appears to be characterized by functionally convergent but taxonomically heterogeneous communities. Distinct microbial configurations may give rise to similar metabolic outputs (e.g., reduced butyrate production, increased endotoxin load or altered BA pools) [60,61]. Microbiome-based stratification may need to focus more on metabolic pathways and community functions than on the presence or absence of specific taxa.

### 3.2. Mechanistic Pathways

#### 3.2.1. Short-Chain Fatty Acids and Other Metabolites

SCFAs, primarily acetate, propionate, and butyrate, are the main end-products of bacterial fermentation of dietary fibers and resistant starches. They act as key mediators of diet–microbiome–host interactions [62,63]. Their biological relevance in MetS extends beyond being “beneficial metabolites,” because SCFAs operate at the interface of (i) epithelial energy metabolism and barrier function, (ii) endocrine signaling, and (iii) immunometabolic regulation [62,63]. Butyrate is the preferred oxidative fuel for colonocytes and supports epithelial respiration, which helps maintain a low-oxygen luminal environment that favors obligate anaerobes and limits expansion of facultative taxa [64]. Thus, SCFAs can contribute to ecosystem stability while simultaneously supporting host mucosal homeostasis [65]. At the barrier level, SCFAs have been shown to enhance epithelial integrity through increased expression and/or assembly of tight junction components (e.g., occludin/claudins/ZO proteins) [66]. In addition, the activation of mucus-associated pathways and the enhancement of antimicrobial defenses and epithelial repair responses act together to reduce intestinal permeability and limit the translocation of pro-inflammatory microbial products [67]. In parallel, SCFAs exert immunomodulatory effects via both receptor-dependent and epigenetic routes, including inhibition of histone deacetylases and signaling through SCFA-sensing receptors expressed on epithelial and immune cells [68], thereby shaping cytokine profiles and supporting regulatory immune phenotypes relevant to the low-grade inflammation characteristic of MetS [69].

Endocrine and metabolic effects are mediated in part by activation of G protein-coupled receptors—notably FFAR2/GPR43 and FFAR3/GPR41—which are expressed on enteroendocrine L cells and other cell types [70,71,72,73]. SCFA signaling in L cells promotes secretion of incretins and satiety hormones, particularly GLP-1 and PYY, linking microbial fermentation to improved postprandial glycemic control, appetite regulation, and gastric emptying dynamics [70,71,72,73]. Beyond gut hormone release, SCFAs can influence systemic metabolism through effects on hepatic lipid handling (including lipogenesis and substrate partitioning), adipose tissue biology, and vascular tone, providing plausible pathways for observed associations with triglycerides, insulin sensitivity, and blood pressure [74]. Mechanistically, these effects are best interpreted as networked outputs of SCFA signaling across tissues (gut–liver–adipose–vasculature), rather than as a single linear pathway [70,71,72,73,74].

In MetS and related phenotypes, multiple cohorts report depletion of SCFA-producing taxa and altered fecal and/or circulating SCFA patterns, although the direction and magnitude of associations are not uniform [75,76]. Importantly, fecal SCFA concentrations reflect the net balance of production, microbial cross-feeding, host absorption, and colonic transit. Therefore, do not always track “SCFA benefit” monotonically across populations; habitual diet composition, sampling matrix (fecal vs. plasma), and analytical methods further contribute to this heterogeneity [75,76]. Despite these measurement caveats, the convergent interpretation across the human and mechanistic literature supports a model in which fiber-poor diets and reduced community capacity for fermentation-related functions are linked to impaired incretin signaling, weakened barrier integrity, and a more pro-inflammatory metabolic milieu, features that align with core pathophysiology of MetS [77].

Trimethylamine *N*-oxide (TMAO), produced from dietary choline, was related to atherosclerosis, CVD events, and mortality [78,79,80]. Moreover, bacteria-driven alterations in branched-chain amino acid metabolism have been linked to insulin resistance, impaired glucose tolerance and type 2 diabetes (T2D) risk [81,82,83]. This association was possibly through effects on mTOR signaling and ectopic lipid accumulation [81,82,83]. Aromatic amino acid-derived indoles and phenolic compounds can influence intestinal barrier integrity, aryl hydrocarbon receptor signaling, incretin secretion, and hepatic inflammation, thereby connecting dietary patterns, microbial metabolism, and NAFLD/MASLD progression [84]. Taken together, these findings support a model in which the gut microbiome functions as a metabolic endocrine organ, producing a complex mixture of small molecules that collectively modulate host metabolic pathways central to MetS.

#### 3.2.2. Bile Acids and FXR/TGR5 Signaling

BAs are not only detergents that facilitate lipid absorption but also endocrine-like signaling molecules that regulate glucose, lipid, and energy homeostasis through nuclear and membrane receptors, particularly the farnesoid X receptor (FXR) and the G protein-coupled receptor TGR5 [85]. BA signaling is inherently microbiome-sensitive because intestinal microbes shape both the composition and signaling potency of the BA pool [86]. Primary BAs synthesized from cholesterol in the liver are conjugated (glycine/taurine) and secreted into the intestine [87], where bacterial bile salt hydrolases (BSH) deconjugate them and enable downstream transformations (e.g., 7α-dehydroxylation, oxidation/epimerization) that generate a diverse set of secondary BAs with distinct receptor affinities [87]. Consequently, changes in microbiome structure and functional capacity translate into shifts in BA diversity, hydrophobicity, and the relative abundance of BA species that act as agonists/antagonists or partial agonists of FXR- and TGR5-driven pathways [88].

Mechanistically, BA–FXR signaling contributes to metabolic regulation through coordinated control of BA synthesis and transport (e.g., feedback inhibition of hepatic BA synthesis), as well as broader effects on hepatic glucose and lipid metabolism [89]. FXR activation influences pathways relevant to MetS, including regulation of gluconeogenesis, lipogenesis, and very-low-density lipoprotein secretion, and it also intersects with enterohepatic signaling through endocrine mediators such as fibroblast growth factor signaling from the gut to the liver (often discussed as a key FXR-linked gut–liver axis mechanism) [90,91]. In parallel, TGR5 activation in metabolically relevant tissues has been linked to energy expenditure and glucose control, in part via effects on thermogenic programs and incretin physiology, providing a plausible route by which BA composition can influence postprandial metabolism and insulin sensitivity [92]. Importantly, BA signaling also integrates with gut barrier and inflammatory biology, because BA species differ in their antimicrobial activity and their capacity to shape microbial niches, while BA receptor signaling can modulate inflammatory tone, features that are highly relevant to chronic low-grade inflammation in MetS [93].

In obesity and MetS, accumulating human and experimental evidence supports a model of dysregulated BA–microbiome crosstalk, characterized by altered BA composition, impaired receptor-mediated signaling, and associations between specific BA signatures, microbial features, and metabolic outcomes such as insulin resistance, dyslipidemia, and hepatic steatosis within the NAFLD/MASLD spectrum [94]. Several studies report that altered BA pools track with hepatic fat content and other cardiometabolic traits, consistent with the concept that BA profiles can serve as both functional readouts of microbiome activity and candidate mediators linking diet to metabolic phenotypes [85]. However, inter-individual variation in diet, medication exposure, and host factors (e.g., liver function, intestinal transit, and enterohepatic circulation dynamics) can influence BA measurements and partially explain heterogeneity across cohorts.

Intervention evidence further supports the therapeutic relevance of this axis. Dietary patterns that restructure the microbiome can shift BA pools, and pharmacologic strategies such as BA sequestrants and receptor-targeting agents (FXR/TGR5 agonists) provide proof-of-concept that modifying BA signaling can influence cardiometabolic risk factors [95,96]. Nevertheless, despite strong biological plausibility, direct causal pathways in humans remain incompletely resolved, and translation to clinical personalization will require studies that link intervention-induced BA changes to downstream receptor signaling, metabolomic outputs, and durable clinical endpoints (e.g., insulin sensitivity, hepatic fat, triglycerides) in well-characterized populations [95,96].

#### 3.2.3. Metabolic Endotoxemia and Low-Grade Inflammation

Metabolic endotoxemia defined as a low-grade elevation of circulating LPS could acts as a trigger for obesity-related insulin resistance and systemic inflammation [97,98]. In rodent models, feeding with a high-fat or Western-type diet increases intestinal permeability and plasma LPS concentrations, and activates TLR4-dependent inflammatory pathways [99,100,101]. In addition, induces weight gain, insulin resistance, and hepatic steatosis; in this regard, antibiotic treatment or genetic disruption of TLR4 signaling attenuates these effects [99,100,101].

**Figure 1:**
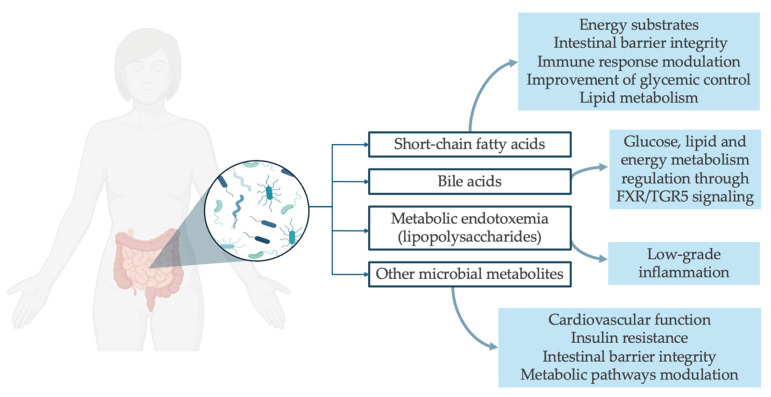
Key mechanistic pathways in gut microbiome–host interactions.

In humans, higher LPS or LPS-binding protein levels are related to abdominal obesity, MetS, and CVD [18,102,103]. Nonetheless, this pathway provides a plausible mechanistic link between Western diets, dysbiosis, increased gut permeability, and systemic inflammatory tone. Figure 1 shows the key mechanistic pathways in gut microbiome–host interactions.

### 3.3. Diet as a Primary Modulator of the Microbiome in Metabolic Syndrome

Among the many determinants of gut microbiome structure, diet is arguably the most powerful and modifiable [104]. Long-term dietary patterns shape the overall community ecology. Moreover, short-term changes in energy intake or macronutrient distribution can induce rapid shifts in microbial composition and function [105]. Diets rich in plant-based foods and fermentable fibers generally increase microbial diversity and the abundance of SCFA-producing species. In contrast, diets high in saturated fat, refined carbohydrates, and low in fiber tend to relate to dysbiosis and pro-inflammatory profiles [106].

The Mediterranean diet has consistently been associated with increased microbial diversity, enrichment of butyrate-producing bacteria, reduced markers of gut inflammation and more favorable metabolic profiles in observational and interventional studies [107,108,109]. Conversely, Western-style dietary patterns have been linked to reduced SCFA production, increased LPS-producing bacteria, and BA profiles related to metabolic dysfunction [110].

Plant-based dietary patterns often promote species that participate in complex carbohydrate fermentation (e.g., *Prevotella* spp.), increase levels of SCFAs, and improve cardiometabolic markers [111]. Beyond macronutrient composition, fiber type and polyphenol content are important modulators of the microbiome. Different fibers (e.g., inulin-type fructans, resistant starch, β-glucans) select for distinct bacterial guilds with varying capacities to produce SCFAs and other metabolites. Polyphenol-rich foods (berries, cocoa, tea, coffee, extra-virgin olive oil) can exert prebiotic-like effects. Increasing these beneficial taxa and SCFA production while their microbial catabolites influence vascular and metabolic pathways [108,109,111].

**Figure 2:**
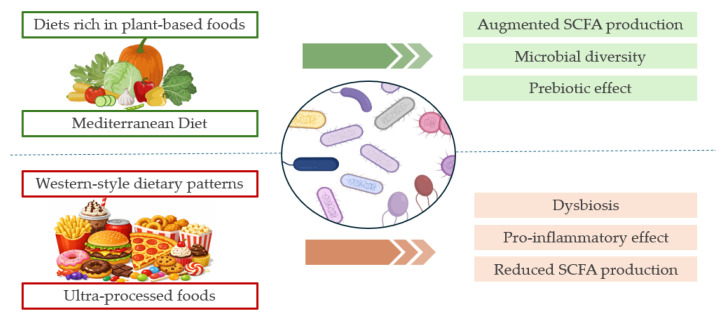
Dietary patterns as modulators of the microbiome.

More recently, attention has turned to ultra-processed foods (UPFs) as a potential disruptor of the diet–microbiome–metabolic axis. UPFs, typically energy-dense, fiber-poor, and rich in additives, are now major contributors to total energy intake in many countries and have been consistently associated with higher risks of obesity, T2D, and CVD [112,113]. Emerging evidence suggests that habitual UPF consumption is linked to reduced microbial diversity, depletion of beneficial commensals, increased gut permeability, and pro-inflammatory microbiota profiles. Providing a plausible mechanistic bridge between UPFs and cardiometabolic risk [113,114]. These effects may be mediated not only by nutrient composition but also by disruption of the food matrix and direct actions of additives on microbial communities and the intestinal barrier [113]. The main modulator effects of dietary patters on the microbiome are represented in Figure 2.

In the context of MetS, these data collectively support the view that diet is both a driver of dysbiosis and a primary lever for microbiome-targeted interventions. Understanding how specific dietary components and patterns reshape microbiome structure and function. Moreover, how these changes translate into metabolic outcomes, provides the foundation for developing microbiome-informed, personalized nutritional strategies in individuals with or at risk of MetS.

## 4. Physical Exercise, Gut Microbiome, and Metabolic Syndrome

### 4.1. Exercise as a Core Component of Lifestyle Management in Metabolic Syndrome

MetS management usually is based on lifestyle changes. Structured physical activity or physical exercise repeatedly showing clinically meaningful benefits across the main MetS domains [115,116,117]. Contemporary syntheses and clinical reviews consistently highlight improvements in insulin sensitivity and glycemic control, blood pressure, atherogenic dyslipidemia, and central and visceral adiposity [115,116,117]. Importantly, these improvements are observed across multiple exercise modalities (aerobic, resistance, and combined training), although the magnitude of benefit typically depends on baseline cardiometabolic risk, adherence, training volume, and whether concomitant dietary energy restriction is present [118]. Importantly, implementation in practice and even in many guideline-adjacent documents still tends to treat them as parallel “pillars” rather than as a single adaptive intervention [115,116,117]. From a mechanistic perspective, this separation is artificial: exercise modifies substrate flux, inflammation, gut motility, bile acid dynamics, and intestinal barrier physiology [115,116,117]. Each of which can plausibly influence microbial ecology and microbial metabolite production, creating a biologically coherent route linking physical activity to gut microbiome-mediated metabolic effects [119].

### 4.2. Effects of Exercise on the Gut Microbiome

#### 4.2.1. Microbiome Composition and Diversity

Physical exercise is frequently associated with higher microbial diversity and detectable changes in community microbial structure. Randomized clinical trials (RCTs) and controlled interventions provide particularly valuable evidence [120]. In adults with overweight/obesity, a 6-month RCT reported a small but significant increase in Shannon diversity in the vigorous-intensity arm and measurable beta-diversity shifts across exercise groups versus control [121]. Notably, the “signal” in such trials is often stronger for community-level structure (beta-diversity) than for single taxa, suggesting that physical exercise may act as a broad ecological perturbation rather than a selective “one-bacterium” intervention [120,121]. Controlled training studies also indicate that exercise can alter the microbiome in ways that depend on baseline adiposity. In one study, compositional and functional changes differed by obesity status and were largely reversible after stopping exercise [122]. This reversibility is an important translational constraint: it implies that microbiome changes may require sustained training to persist, and that studies with short-term interventions or poor adherence are likely to underestimate true effects. At the taxonomic level, many studies and reviews describe enrichment of taxa often linked to SCFA production, including butyrate-associated genera (e.g., *Faecalibacterium* and *Roseburia* in some cohorts). However, results are heterogeneous and not uniformly replicated, likely reflecting differences in participant characteristics, exercise prescription (aerobic vs. resistance vs. type), study duration, diet control, and sequencing/analytic pipelines [123,124,125,126]. For example, some interventions report increases in taxa typically considered “beneficial” in metabolic health (often within butyrate-producing guilds), whereas other studies show minimal genus-level changes despite clear physiological improvements, implying that the functional output of the microbiome may shift even when taxonomy appears stable [123,124,125,126]. A critical interpretation is that physical exercise effects on taxonomy may be contingent on the dietary substrate environment, as without adequate fermentable fiber intake, expansion of saccharolytic/butyrate-producing communities may be constrained, which could partially explain inconsistent taxonomic findings across cohorts with different habitual diets [121,122,123,124].

#### 4.2.2. Microbial Metabolites and Host Physiology

Mechanistically, exercise–microbiome links are increasingly interpreted through the lens of microbial metabolites [127]. SCFAs are a leading candidate pathway because they connect microbial fermentation to gut barrier integrity, inflammatory tone, and metabolic regulation [127]. In controlled human training, exercise increased fecal SCFAs in lean participants and exercise-related changes in microbial functional potential aligned with shifts in SCFA-producing capacity [122]. This is consistent with a model in which physical exercise increases intestinal transit dynamics and substrate availability to distal colonic fermenters, while also lowering systemic inflammation, conditions that may favor SCFA-producing consortia and/or their metabolic activity [122]. Broader reviews converge on the idea that exercise can support SCFA-related functionality and improve gut barrier and systemic metabolic signaling [124,128,129], although the magnitude and durability of these effects likely depend on baseline metabolic health and the sustainability of the activity pattern [124,128,129].

However, an important nuance is that higher fecal SCFAs do not necessarily imply higher host absorption or beneficial signaling, because fecal concentrations reflect the balance between production, host uptake, and transit time [130,131]. Therefore, future studies should triangulate fecal SCFAs with circulating SCFAs, targeted metabolomics, and host signaling readouts (e.g., GLP-1/PYY, inflammatory markers) to strengthen mechanistic inference [132,133]. Beyond SCFAs, exercise may influence microbial pathways linked to branched-chain amino acid metabolism, lactate cross-feeding, and aromatic amino acid derivatives, which are increasingly implicated in insulin sensitivity and inflammatory tone [134]. However, evidence remains less consistent than for SCFA-related functions and requires more standardized functional profiling [135].

Exercise may also influence BA profiles indirectly through changes in the gut microbiome and host metabolism [136,137]. This could be carried out by FXR/TGR5-mediated signaling pathways implicated in lipid and glucose homeostasis. The biological plausibility of microbiome-driven BA modulation as a metabolic lever is well supported by authoritative reviews of BA–microbiome–receptor biology [136,137]. From a physiological standpoint, exercise can alter BA circulation through effects on hepatic metabolism, intestinal motility, and enterohepatic cycling. These host-driven changes can then feed back to the microbiome because of BA composition and concentration shape microbial selection pressures and antimicrobial constraints [138]. Nevertheless, BA outcomes are particularly sensitive to sampling context (fasting vs. postprandial), diet composition, and analytical platform [139]. Thus, discrepancies across studies may reflect methodological rather than biological differences, emphasizing the need for harmonized BA profiling in exercise–microbiome research [119,127].

Collectively, these observations support a synergy model. Diet provides the substrate environment for microbial metabolism, while exercise can reshape intestinal physiology and microbial ecology, together amplifying metabolic benefits [124,140,141]. This synergy framework predicts that the largest microbiome-mediated benefits occur when physical exercise is paired with dietary patterns that provide fermentable substrates (e.g., Mediterranean-style, fiber-rich diets), whereas exercise in a low-fiber dietary context may yield smaller or more variable microbiome shifts [142].

### 4.3. Exercise–Microbiome Interventions in Metabolic Syndrome and Obesity

Intervention evidence in obesity/MetS-adjacent populations increasingly supports the idea that exercise, alone or combined with diet, can remodel gut ecology [143]. However, also makes clear that effects are often subtle, context-dependent, and require careful interpretation [143]. RCTs in adults with overweight/obesity demonstrate that structured exercise can shift beta-diversity and inferred functional potential, even when genus-level changes are limited [121,143,144]. This pattern suggests that exercise may primarily affect microbial “activity states” (functional capacity/expression) rather than producing large, consistent taxonomic turnover—an interpretation aligned with the observation that physiological improvements can occur in parallel with modest compositional changes [121,143,144]. Complementary controlled trials show that exercise-induced microbial changes can differ by obesity status and may revert when training stops. Moreover, they highlight the importance of adherence and long-term maintenance for durable microbiome modulation [122,144]. From a clinical perspective, this indicates that microbiome modulation should not be framed as an automatic consequence of prescribing exercise; it depends on sustained behavior change and may require complementary dietary design to support ecological stability [122,144].

Beyond exercise-only designs, combined lifestyle interventions provide a pragmatic template closer to real clinical care. In PREDIMED-Plus, a 1-year lifestyle intervention incorporating an energy-restricted Mediterranean diet and physical activity was associated with gut microbiota changes linked to SCFA-producing bacteria [140]. This is particularly relevant because it reflects a real-world intervention package where diet provides fermentable substrate and exercise may reinforce barrier and metabolic improvements, which are conditions expected to favor SCFA-related ecology [140]. A more recent RCT in the same framework has extended these observations to the gut metabolome and microbiota in relation to cardiometabolic risk factors [145]. The addition of metabolomic readouts is important because it enables testing whether microbiome changes translate into functional chemical outputs that plausibly mediate cardiometabolic improvements, rather than relying on taxonomy alone [145]. In metabolically compromised patients (NAFLD with prediabetes), a four-arm randomized controlled trial showed that the combined aerobic exercise + diet intervention was associated with diversified and stabilized keystone taxa and that baseline microbial network properties could help predict individual liver-fat response [141]. This is an important proof-of-concept for microbiome-informed stratification [141]. Critically, such results suggest that microbial network features (i.e., community connectivity/keystones) may provide more clinically useful “response biomarkers” than single taxa, because they capture ecological stability and resilience—properties likely relevant to long-term metabolic maintenance [141]. At the same time, network metrics can be sensitive to sequencing depth, compositionality, and analytic choices. Therefore, replication across cohorts and standardized network pipelines are essential before these approaches can be translated into clinical tools [141].

Taken together, these trials suggest three clinically relevant messages: (i) exercise can influence the gut microbiome in humans, (ii) the most translational signals may lie in functional/metabolite readouts and network properties rather than single taxa, and (iii) heterogeneity of response is not noise to be averaged away but a feature that precision lifestyle strategies should aim to explain and harness [122,140,141,145].

## 5. Microbiome-Informed Personalized Nutrition in Metabolic Syndrome

### 5.1. Evidence from Observational Studies

A consistent body of observational evidence indicates that dietary patterns linked to lower MetS risk [146]. In a large prospective analysis, Mediterranean-style diet adherence related to cardiometabolic outcomes varied according to baseline microbial composition [147]. This implying that a “one-size-fits-all” dietary recommendation may yield heterogeneous benefit partly due to differences in microbial functional potential (e.g., carbohydrate utilization, BA transformations, and other microbially mediated metabolic routes) [147].

### 5.2. Intervention Studies Targeting the Microbiome in Metabolic Syndrome

Whole-diet interventions. Controlled dietary interventions provide stronger evidence that shifting dietary pattern can induce coordinated changes in gut microbiome structure and metabolic readouts relevant to MetS [148]. For example, switching to a Mediterranean diet has been shown to lower plasma cholesterol and reshape both the gut microbiome and metabolome [149]. Moreover, the diet-induced metabolic changes co-varied with specific microbial taxa and microbial metabolic outputs (including BA-related features) [149]. More “enhanced” Mediterranean variants (e.g., Green-MED) have further supported that microbiome features may partially mediate improvements in cardiometabolic risk markers. This reinforce the need to move from descriptive microbiome changes to mechanistically anchored mediators [150].

Specific components/supplements. A major strategy has been to increase fermentable substrates (prebiotics) and target SCFA production capacity. High-fiber dietary interventions can selectively promote SCFA-producing organisms and improve glycemic control in humans [151,152]. Resistant starch has also emerged as a promising substrate. In an 8-week supplementation trial in individuals with excess body weight reported improvements in insulin resistance alongside microbiome shifts, with *Bifidobacterium adolescentis* highlighted as a candidate taxon linked to benefit [153]. In a proof-of-concept randomized trial, pasteurized *Akkermansia muciniphila* supplementation in overweight/obese insulin-resistant adults was safe and showed directionally favorable metabolic signals versus placebo, bringing the field closer to organism-level, mechanism-driven interventions [154]. Evidence for conventional probiotics/synbiotics in MetS remains mixed but suggests modest improvements in selected cardiometabolic traits in meta-analytic summaries, tempered by strain specificity, short follow-up, and variability in endpoints and co-interventions [155,156]. Polyphenols, omega-3, and multi-component formulations are also being explored for microbiome modulation with cardiometabolic relevance. However, attribution to a single component is often limited by combined interventions and heterogeneous microbiome methods [157,158,159].

Advanced microbiome-based therapies. Fecal microbiota transplantation provides an informative “causal probe” in MetS. In a seminal randomized study, lean-donor intestinal microbiota infusion increased insulin sensitivity at 6 weeks in male recipients with MetS, with corresponding changes in microbial composition [160]. Subsequent work underscored the transient nature of benefit and the importance of baseline recipient microbiome configuration in predicting response, emphasizing that “donor–recipient matching” and ecological engraftment constraints are central barriers to reliable translation [161]. Newer trials testing adjunct strategies (e.g., fiber to support engraftment) reflect a pragmatic evolution toward combined, ecology-supportive protocols, but durability and scalability remain unresolved [162]. Table 1 summarizes the main effects of interventional studies regarding microbiome, exercise, and diet in patients with MetS.

### 5.3. Trials Explicitly Using Microbiome in Personalized Nutrition Algorithms

The most mature “microbiome-informed personalization” paradigm has been the prediction of postprandial responses using integrated clinical, dietary, and microbiome features [28]. A landmark study demonstrated that machine-learning models incorporating microbiome data can predict individualized postprandial glycemic responses, and that algorithm-guided dietary advice can improve glycemic control compared with standardized guidance in controlled settings [163,164].

More recently, intervention studies have started to test “microbiome-aware” or multi-kingdom microbiome personalization approaches in dysglycemia/prediabetes—an adjacent phenotype tightly linked to MetS trajectories. For instance, microbiome features (gut and/or oral) have been integrated into dietary intervention frameworks, highlighting both predictive potential and the practical need for interpretable, clinic-friendly decision rules [165,166,167]. Overall, these trials position the microbiome not merely as a correlational marker but as a measurable layer that can (i) stratify responders, (ii) guide selection among dietary options (e.g., fiber types), and (iii) provide intermediate endpoints for monitoring adherence and biological effect—yet external validation across populations, labs, and diet cultures remains a key translational requirement.

### 5.4. Effects on Related Comorbidities

*Type 2 diabetes/prediabetes*. Diet–microbiome interventions in dysglycemia provide some of the strongest proof-of-concept microbial functional targeting (especially SCFA-related ecology) [168]. However, generalization to broader MetS populations requires caution given differences in baseline phenotype and medication exposure [151]. Personalized nutrition algorithms leveraging the microbiome further support the feasibility of “response-guided” dietary prescriptions for glycemic control [169].

*NAFLD/MAFLD*. Microbial transformations of BA and signaling through FXR/TGR5 integrate with host lipid/glucose metabolism and inflammatory tone. This offers mechanistic targets for microbiome-informed dietary strategies [88,96,170]. Clinical lifestyle trials in NAFLD have reported microbiome rearrangements alongside improvements in hepatic steatosis-related measures, supporting the plausibility of microbiome-linked pathways in liver outcomes, although causal mediation remains incompletely established [171,172].

*Cardiovascular disease*. Microbiome-mediated metabolites provide a direct bridge from habitual diet to vascular risk biology [21]. The choline/carnitine/TMAO pathway, in particular, has been mechanistically tied to atherosclerosis-related processes and associated with cardiometabolic outcomes in prospective settings [173]. This makes it a prime example of a diet–microbiome–metabolite axis with potential utility for risk stratification and targeted dietary modification [174,175,176].

## 6. Clinical Translation and Implementation Challenges

### 6.1. Heterogeneity of Response and Metabolic Phenotypes

MetS is not a single biological entity but a syndrome-level label that aggregates distinct underlying pathophysiologies [177]. In practice, patients often cluster into partially overlapping tissue-dominant metabolic phenotypes [178]. Adipose dysfunction, hepatic insulin resistance/steatosis, or skeletal muscle insulin resistance may predominate [179], each with different biomarker profiles and potentially different dietary leverage points (e.g., macronutrient quality/quantity, energy restriction, or dietary fat composition) [180,181]. Evidence from long-term dietary interventions supports this concept: in the CORDIOPREV-DIAB randomized trial, baseline liver vs. muscle insulin-resistance phenotypes modified metabolic responses to different diet patterns over follow-up [182], illustrating why “one-size-fits-all” advice can yield heterogeneous results in MetS-like populations [183].

This heterogeneity is further amplified by the gut microbiome, where inter-individual differences in community structure and functional capacity can meaningfully shape metabolic responses to the same foods [184]. Large, deeply phenotyped studies of postprandial metabolism demonstrate that person-specific factors contribute to variability in glycemic and lipemic responses [17,35,184]. This reinforces the idea that microbiome-informed stratification could help explain non-response and guide more targeted dietary prescriptions.

### 6.2. Methodological Challenges

Microbiome findings can vary substantially with choices across the analytic chain [185], sequencing platform and library preparation, reference databases, taxonomic/functional profiling tools, normalization, contaminant handling, and statistical models for differential abundance [186]. Comparative evaluations show that different differential abundance methods and pipelines can produce meaningfully different “discoveries” on the same underlying datasets [185,187,188]. These aspects directly impact biomarker credibility and downstream clinical claims. To address this, the field has increasingly emphasized standardized reporting and transparent methods [189]. The STORMS reporting guidelines were developed specifically to improve comparability and interpretability across human microbiome studies [189].

Many precision nutrition and microbiome intervention studies remain limited by small sample sizes, short follow-up, inconsistent outcome definitions, and limited replication and external validation [190]. Workshop-based and systematic syntheses highlight that robust translation will require better-powered studies, harmonized endpoints, and prospective validation in independent cohorts before clinical adoption can be justified [191,192].

### 6.3. Practical and Ethical Aspects

Multi-omics profiling and continuous digital monitoring can be costly and logistically complex. In contrast, the clinical workforce is not uniformly trained to interpret omics-derived outputs or machine learning (ML)-based predictions [193,194]. Some reviews on the intersection of digital health and personalized nutrition repeatedly identify the need for user-friendly interfaces. Here, the important variables are related to clinical decision support, and clinician education so that precision recommendations are interpretable, actionable, and aligned with standard care pathways [30,193,195].

Ethically, the combination of omics data and high-frequency digital phenotypes (wearables and apps) raises non-trivial concerns around consent, data governance, secondary use, and privacy [196]. Recent reviews of AI-driven precision nutrition and digital-health ecosystems emphasize that privacy safeguards, transparency, and regulatory alignment must be treated as core design requirements rather than afterthoughts [196,197,198]. Particularly as commercial platforms increasingly mediate data capture and recommendation delivery.

### 6.4. Equity and Generalizability

Public microbiome resources and many precision nutrition datasets remain disproportionately drawn from Western, high-income settings [199]. This limits the portability of microbiome biomarkers and prediction models to populations with different ancestries, food environments, infectious exposures, and sociocultural contexts [200]. Quantitative audits of public microbiome data demonstrate strong geographic skew. Recent perspectives and large-scale efforts explicitly argue that underrepresentation constrains discovery and risks widening health disparities [201]. The field needs study designs that deliberately include diverse diets and contexts, invest in regional research capacity, and validate tools across settings—so that “precision nutrition” does not become “precision for the few”.

### 6.5. How Clinicians Can Use Microbiome Data Today and Next Steps for Implementation

At present, clinically actionable use of microbiome science remains uneven across indications. The strongest evidence base and clearest care pathways are concentrated in selected gastrointestinal settings [19], particularly with recurrent *Clostridioides difficile* infection, where fecal microbiota-based therapies and microbiota restoration strategies have demonstrated clinical benefit and are increasingly reflected in clinical guidance and pivotal trials [202,203,204]. In contrast, for MetS, most outputs from 16S rRNA gene sequencing or metagenomics remain insufficiently validated for routine decision-making. This is emphasized by recent consensus efforts urging caution when translating microbiome test reports into clinical recommendations without rigorous validation and clear clinical action thresholds [205]. Consequently, when microbiome testing is obtained in MetS-like populations, results should generally be interpreted as hypothesis-generating and contextualized alongside diet quality, medication exposures (including antibiotics and acid-suppressing drugs), adiposity distribution, hepatic steatosis markers, and glycemic patterns, rather than used as stand-alone determinants of dietary prescriptions [205].

A feasible implementation pathway in cardiometabolic care requires moving beyond descriptive “dysbiosis” labels toward reproducible, function-centered outputs that can be audited clinically. First, microbiome measurement must become more reproducible through harmonized pre-analytics, sequencing, and bioinformatic workflows, and through transparent reporting standards; adoption of structured reporting frameworks such as STORMS is a necessary foundation to improve comparability and interpretability across human studies [189]. Second, methodological choices across the analytic chain can materially change results; comparative evaluations show that different differential-abundance methods and pipelines can yield meaningfully different “discoveries” on identical datasets, directly affecting biomarker credibility and downstream clinical claims [186]. Third, translation will depend on demonstrating incremental value over standard risk stratification using clinically meaningful endpoints (e.g., glycemic trajectories, blood pressure, lipids, hepatic fat, and weight maintenance), with external validation before adoption—an approach aligned with expert recommendations for clinical microbiome testing and interpretation [205]. Fourth, implementation should prioritize clinician-facing decision support that produces interpretable. Moreover, guideline-compatible recommendations rather than long lists of taxa reflect the broader consensus that clinical usefulness depends on actionable outputs with explicit uncertainty and validated thresholds [205].

Finally, feasibility, privacy, and equity must be treated as core design requirements. Public microbiome datasets are geographically skewed toward high-income settings, which constrains generalizability and risks widening disparities if biomarkers and models are deployed without validation in diverse ancestries, diets, and environments [201]. At the same time, microbiome-based precision approaches increasingly intersect with sensitive omics and digital phenotypes, raising privacy and governance challenges that require robust safeguards, particularly when data are handled through commercial or cross-institutional pipelines [206]. Together, these considerations reinforce that near-term progress in MetS will be driven less by additional associative findings and more by standardized measurement, rigorous validation, equity-conscious cohort building, and privacy-preserving translational infrastructure [186,189,201,205,206].

## 7. Future Directions

The future of precision nutrition lies in a decisive shift away from isolated, single-layer associations toward integrated, mechanism-informed biological signatures that are reproducible across cohorts and analytically robust [207]. In microbiome research, this transition requires moving beyond descriptive profiling to the coordinated integration of complementary data layers. Shotgun metagenomics provides insight into taxonomic composition and functional potential, but when combined with metatranscriptomics, metaproteomics, and metabolomics to unravel microbial functions, they can be meaningfully linked to host cardiometabolic pathways that are actionable through diet [208]. The greatest gains are likely to come from analyses that explicitly integrate microbial and host-derived omics, such as circulating metabolomics, to bridge microbial activity with systemic metabolic regulation.

Comprehensive reviews of multi-omic integration consistently underline this promise, while also issuing an important caveat: integration alone is not sufficient. Without rigorous standardization, harmonized analytical pipelines, robust quality control, and independent validation, multi-omic model risk being complex without being reliable [209]. Establishing shared methodological frameworks will therefore be essential if microbiome-informed signatures are to move from exploratory research into clinically meaningful tools.

In parallel, ML and AI are rapidly becoming central to precision nutrition research. However, the primary barrier to translation is no longer predictive performance, but interpretability and clinical trust. Seminal studies demonstrating the prediction of individual postprandial responses illustrate the transformative potential of data-driven models, while recent syntheses of the field reveal a fast-growing AI ecosystem accompanied by persistent shortcomings, including limited generalizability across populations, inconsistent benchmarking practices, and insufficient attention to transparency, equity, and deployment in real-world settings [17,210]. In response, there is a clear shift toward explainable approaches—such as feature attribution, constrained modeling, and model simplification—that prioritize clinical interpretability, facilitate auditing, and support patient-centered decision-making rather than opaque “black-box” predictions [211].

Equally important is the adoption of a life-course perspective. Early life represents a critical window of developmental plasticity for both host metabolism and the gut microbiome. Accumulating evidence links early microbial configurations and microbial-derived metabolites to metabolic phenotypes later in life, suggesting that cardiometabolic risk may be shaped long before clinical disease becomes apparent [212]. This recognition is driving a new generation of cohort studies that begin in pregnancy or infancy, incorporate repeated multi-omic sampling and explicitly examine pediatric cardiometabolic trajectories alongside potential intergenerational influences, both biological and social [213]. Together, these studies reinforce the concept that early-life exposure can durably imprint microbial and metabolic features, strengthening the rationale for prevention-oriented precision nutrition strategies initiated well ahead of overt disease [214,215].

Finally, truly “precise” nutrition must move beyond biology alone to incorporate behavioral, psychological, and environmental determinants that shape both physiological responses and long-term adherence. Sleep quality, physical activity, psychosocial stress, and socioeconomic context all influence dietary exposures and metabolic outcomes [216]. In addition, circadian alignment and meal timing are increasingly recognized as mechanistically relevant regulators of metabolism, with potential interactions across microbial and host pathways [217]. Digital health technologies, including wearables, continuous glucose monitoring, and high-resolution dietary assessment tools, offer practical avenues to capture these dynamic factors and support adaptive, context-aware interventions. At the same time, their integration demands careful validation, governance, and ethical oversight to ensure that increased data complexity leads to better decisions and broader benefit, rather than confusion or widening health disparities [218].

## 8. Conclusions

The gut microbiome has emerged as a key biological link between diet, lifestyle, and metabolic health in MetS, helping to explain why individuals often respond so differently to the same dietary advice. By shaping energy harvest, glucose and lipid metabolism, BA signaling, gut barrier integrity, and systemic inflammation—largely through the actions of microbial metabolites—the microbiome provides a biologically plausible framework for more personalized interventions. A growing body of evidence shows that diet and physical activity can be used to modulate microbiome composition and function in ways that meaningfully influence cardiometabolic risk, positioning microbiome-informed personalized nutrition as a natural evolution beyond “one-size-fits-all” approaches.

At the same time, this field remains in its early stages. Much of the current evidence comes from relatively small, short-term, and methodologically heterogeneous studies. Encouragingly, research is now shifting toward mechanistic studies, controlled interventions, and questions of real-world implementation. With stronger long-term evidence, greater methodological standardization, and the development of interpretable and scalable tools, microbiome-informed diet and exercise strategies have the potential to refine the prevention and management of MetS and deliver more precise, durable, and equitable reductions in cardiometabolic risk.

## Figures and Tables

**Table 1:** Effect of intervention studies in the context of MetS.

Physical Exercise	
Structured exercise interventions	Changes in beta-diversity and functional potential
Combined with energy-restricted Mediterranean diet	Increased of SCFA-producing bacteria
Aerobic exercise and diet intervention	Diversified and stabilized keystone taxa in patients with NAFLD and prediabetes
Dietary interventions	
Whole-diet interventions	Changes in gut microbiome structure and metabolic readouts
Specific components or supplements	
High-fiber dietary/prebiotics	Increase in SCFA-producing organisms and improvement of glycemic control
Resistant starch	Reduction in insulin resistance alongside microbiome shifts (*Bifidobacterium adolescentis*)
Akkermansia muciniphila	Favorable metabolic signals
Fecal microbiota transplantation	Changes in microbial composition and increased insulin sensitivity

*Abbreviations.* NAFLD, non-alcoholic fatty liver disease; SCFAs, short-chain fatty acids.

## Data Availability

Not applicable.
